# Overexpression of 15-lipoxygenase-1 in oxygen-induced ischemic retinopathy inhibits retinal neovascularization via downregulation of vascular endothelial growth factor-A expression

**Published:** 2012-11-30

**Authors:** Zhi Li, Tao He, Ke Du, Yi-Qiao Xing, Ying Yan, Zhen Chen, Hao Zhang, Yin Shen

**Affiliations:** 1 Eye Center, Renmin Hospital of Wuhan University, Wuhan, China; 2 Department of Ophthalmology, Xiangyang Center Hospital, Xiangyang, China; 3 Department of Oncology, Xiangyang Center Hospital, Xiangyang, China; 4 Department of Ophthalmology, Wuhan General Hospital of Guangzhou Military, Wuhan, China; 5 Department of Cardiothoracic Surgery, Xiangyang Center Hospital, Xiangyang, China

## Abstract

**Purpose:**

15-Lipoxygenase-1 (15-LOX-1) plays an important role in regulating angiogenesis, but the mechanism to date is controversial, even contradictory. The goal of our study was to investigate whether 15-LOX-1 plays a role in inhibiting retinal neovascularization (RNV) in a mouse model of oxygen-induced retinopathy (OIR) and the underlying mechanism.

**Methods:**

Experiments were performed using retinas from a mouse model of OIR that was treated with and without intravitreous injection of adenoviral-15-lipoxygenase-1 (*Ad-15-LOX-1)* or adenoviral-green fluorescence protein (*Ad-GFP*) at postnatal day 12 (P12). At P17, the efficacy of the gene transfer was assessed with immunofluorescence staining. RNV was evaluated with fluorescein angiography on flatmounted retinas and quantified by counting the preretinal neovascular cells. Expression of 15-LOX-1 and vascular endothelial growth factor-A (VEGF-A) were determined with real-time PCR and western blot.

**Results:**

RNV during OIR was associated with decreased 15-LOX-1 expression, and retinal 15-LOX-1 levels were negatively correlated with the progression of RNV. In the intravitreous injected *Ad-15-LOX-1* mice with OIR, retinal 15-LOX-1 expression was significantly increased at the protein and mRNA levels at P17. 15-LOX-1 expression was clearly demonstrated, primarily in the outer plexiform layer, inner nuclear layer, and ganglion cell layer retinas, five days after gene delivery. Fluorescein retinal angiography and quantification of the preretinal neovascular cells demonstrated that RNV was significantly inhibited. Meanwhile, the expression levels of VEGF-A were significantly decreased at the transcriptional and translational levels.

**Conclusions:**

Our results suggest that overexpression of 15-LOX-1 inhibits RNV in a mouse model of OIR via downregulation of VEGF-A expression, and 15-LOX-1 may be a novel therapeutic target for ocular neovascularization diseases.

## Introduction

Retinal neovascularization (RNV) is a characteristic pathologic finding of many ocular diseases and a major cause of the blindness associated with ischemic retinopathy such as diabetic retinopathy, retinal vein occlusion, and retinopathy of prematurity. RNV is controlled by two counter-balancing systems of proangiogenic factors and antiangiogenic factors [1]. However, under some pathological conditions, the balance is disrupted by enhanced production of proangiogenic factors and/or downregulation of antiangiogenic factors [2]. The exact mechanism underlying the pathogenesis of RNV is not yet well understood.

Lipoxygenases (LOXs) constitute a heterogeneous family of lipid peroxidizing enzymes that catalyze the stereoselective dioxygenation of polyunsaturated fatty acids to their corresponding hydroperoxy derivatives [3,4]. In mammals, LOXs are categorized regarding their positional specificity of arachidonic acid oxygenation into 5-, 8-, 12-, and 15-LOXs [3-5]. Within the 15-LOXs, two isoforms have been identified [6]. 15-LOX-1 is mainly expressed in reticulocytes, eosinophils, immature red blood cells, macrophages, airway epithelial cells, and skin [7-9]. 15-LOX-2 has limited tissue expression in the prostate, lung, skin, and cornea [10]. In terms of enzymatic characteristics, 15-LOX-1 preferentially metabolizes linoleic acid to 13-(S)-HPODE and 13-(S)-HODE, but also metabolizes arachidonic acid to 15-(S)-HPETE and 15-(S)-HETE [8,11]. However, 15-LOX-2 metabolizes arachidonic acid to 15-(S)-HPETE and 15-(S)-HETE, and metabolizes linoleic acid poorly [6]. 15-LOX-1 is involved in many pathological conditions, such as cell differentiation, inﬂammation, atherogenesis, and carcinogenesis [12].

The expression and function of 15-LOX-1 have been studied in endothelial cells, smooth muscle cells, and monocytes [8], and 15-LOX-1 has been shown to play key roles in vascular remodeling [4] and the progression of atherosclerosis [4,13-15]. 15-LOX-1 and its metabolites have been implicated for their role in angiogenesis. 15-LOX-1 activates peroxisome proliferator-activated receptor (PPAR)-γ through 13-(S)-HODE [16,17], and PPAR-γ activation inhibits angiogenesis [18,19]. 15-(S)-HETE exerts a proangiogenic action while 15-(S)-HPETE impairs the angiogenic process [20]. However, thus far, the role of 15-LOX-1 in angiogenesis remains controversial [21], and the underlying mechanisms of the effect of 15-LOX-1 in angiogenesis remain unclear.

Vascular endothelial growth factor (VEGF) has been demonstrated to play an important role in ocular neovascularization [22,23]. Increased levels of VEGF have been detected in the retina and vitreous humor of patients with ischemic retinopathy [24,25], as well as in animal models of ocular neovascularization [26-28]. In mammals, the VEGF family consists of seven members: VEGF-A, VEGF-B, VEGF-C, VEGF-D, VEGF-E, VEGF-F, and placental growth factor (PIGF) [29-33]. Of these factors, VEGF-A seems to be the most important growth factor in angiogenesis [23,34].

The role of 15-LOX-1 in the development of RNV and the relationship between 15-LOX-1 and VEGF-A in RNV have not been well investigated. The goal of this study was to explore the changes in 15-LOX-1 expression during RNV and determine whether 15-LOX-1 activity impacts RNV going through changes in the level of VEGF-A.

The current study suggests, for the first time, that oxygen-induced retinopathy (OIR) is associated with decreased 15-LOX-1 expression. Intravitreal injection of *Ad-15-LOX-1* significantly inhibited RNV and downregulated VEGF-A expression in OIR. Thus, 15-LOX-1 gene transfer is a potential new strategy for treating ocular neovascularization diseases.

## Methods

### Recombinant adenoviral vector construction

Two recombinant adenoviral vectors based on the pDC315-EGFP vector (purchased from Shanghai GeneChem Co. Ltd) were constructed expressing the mouse 15-LOX-1 gene (*Ad-15-LOX-1*) and the green fluorescence protein gene (*Ad-GFP*; control), respectively, under the control of the mouse cytomegalovirus (CMV) promoter. The stock solutions of *Ad-15-LOX-1* and *Ad-GFP* were 1.25×10^11^ and 2.50×10^11^ plaque formation unit (PFU)/ml, respectively. A working solution was prepared to make 1 μl of vehicle containing approximately 1.0×10^9^ PFU.

### Animal model of oxygen-induced ischemic retinopathy

Pregnant female C57BL/6J mice were provided by the Laboratory Animal Center of Wuhan University. All experiments were conducted in accordance with the Association for Research in Vision and Ophthalmology (ARVO) Resolution on the Care and Use of Laboratory Animals. The oxygen-induced ischemic retinopathy model was performed on C57BL/6J mice according to Smith et al.’s method [35] with some modifications [36]. Briefly, at postnatal day 7 (P7), pups with their mothers were exposed to hyperoxia (75±2% O_2_) for five days (P7–P12) and then returned to normoxia (room air) for five days. Neovascularization occurred when the mice returned to normoxia and peaked at postnatal day 17 (P17). The experiments were randomly divided into four groups [1]: the normal control group (the normal group) [2], the untreated OIR group (the OIR group) [3], the OIR treated with *Ad-15-LOX-1* group (the 15-LOX-1 group) [4], and the OIR treated with *Ad-GFP* group (the GFP group). In the normal group, newborn mice were maintained in room air from P0 to P17. In the OIR group, OIR was induced in C57BL/6J pups from P7 to P17. In the 15-LOX-1 group and the GFP group, the OIR-induction protocol was used. The mice were given an intravitreal injection of 1 μl of vehicle containing approximately 1.0×10^9^ PFU of *Ad-15-LOX-1* or *Ad-GFP* at P12, and then returned to normoxia for five days. Mice in the normal group and the OIR group were anesthetized with ketamine hydrochloride (100 mg/kg bodyweight), and then were killed at the 7th, 9th, 12th, 14th, 17th, and 21st postnatal day to detect 15-LOX-1 expression during RNV with real-time PCR and western blot. Meanwhile, mice in all four groups were euthanized at P17, and one retina was collected from each mouse for biochemical assays while another eye was harvested as a whole for morphological study.

### Intravitreous injections of recombinant adenoviral vectors in mice

Intravitreous injections were administered with a Harvard pump microinjection apparatus and pulled glass micropipets, as previously described [37,38]. Briefly, under a dissecting microscope, the sharpened tip of a micropipette was passed through the sclera, just behind the limbus into the vitreous cavity. Each micropipet was calibrated to deliver 1 μl of vehicle containing approximately 1.0×10^9^ PFU of *Ad-15-LOX-1* or *Ad-GFP*.

### Fluorescence microscopy after intravitreal injections of *Ad-15-LOX-1*

To examine the expression of *Ad-15-LOX-1* in retinas, five days after intravitreal injections of the *Ad-15-LOX-1* or *Ad-GFP* vector, the mice were euthanized, and the eyes were used to make cryosections. The retinas were prepared following the protocol previously described [39]. Briefly, the retinas were immediately fixed in 4% paraformaldehyde in 0.1 M phosphate buffer (PB, pH 7.4) for 20 min and chilled sequentially in 10% (w/v), 20%, and 30% sucrose in 0.1 M PB at 4 °C overnight. The retinas were then embedded in optimal cutting temperature (OCT) compound (Miles Inc., Elkhart, IN), frozen with liquid nitrogen, and sectioned vertically at 14 μm thickness on a freezing microtome (Leica, Nussloch, Germany). The sections were mounted on gelatin-coated slides. Immunofluorescence staining was performed as described previously [40] with some modifications. Briefly, deparaffinized and rehydrated cryosections were exposed to hydrogen peroxide to eliminate endogenous peroxide activity. The sections were then permeabilized for 30 min using PBS/0.4% Triton X-100 followed by blocking in PBS/5% BSA for 20 min. After 3×10 min washes with PBS, the sections were incubated with a mouse monoclonal antibody to 15-LOX-1 (5 μg/ml, Abcam, Cambridge, MA) at 4 °C overnight. The sections were then rinsed in PBS/0.2% Triton X-100 and incubated with a Cy3-conjugated goat anti-mouse immunoglobulin G (1:500, Jackson Immunoresearch Lab. Inc., West Grove, PA) for 1 h. We omitted a primary antibody (15-LOX-1) and a secondary antibody for the control experiments. The stained sections were rinsed again and mounted in a mounting medium containing 4', 6-diamidino-2-phenylindole (DAPI). The slides were viewed at 400× magnification with a Nikon Eclipse Ti-SR fluorescence microscope (Nikon, Tokyo, Japan).

### Fluorescein retinal angiography assessment of new vessel formation and avascular area changes

Fluorescein retinal angiography was performed as previously described [35,41]. Briefly, mice were anesthetized and perfused via the left ventricle with 50 mg/ml high molecular weight (2×10^6^) fluorescein isothiocyanate-dextran (Sigma). The mice were immediately euthanized, and the eyes were enucleated and fixed in 4% paraformaldehyde for 3 h. The retina was flatmounted on a gelatin-coated slide. Quantification of RNV was performed as described previously [42,43]. Briefly, images of the retina were taken at 40× magnification on an Olympus BX41 fluorescence microscope (Tokyo, Japan) and imported into Adobe Photoshop. Neovascular tuft formation was quantified by comparing the number of pixels in the affected areas with the total number of pixels in the retina. The avascular area in the retina was measured in the same way. RNV and the avascular area were measured blind to the identity of the sample.

### Quantification of preretinal neovascular cells

Seventeen-day-old C57BL/6J mice were anesthetized and perfused transcardially with PBS. The eyes were fixed in Bouin’s solution for 4 h and stored in 70% ethanol. After fixation, the eyes were embedded and cross-sectioned vertically through the center of the cornea and optic nerve. To quantify the preretinal neovascular cells, retinal structures were analyzed on 4 μm hematoxylin and eosin–stained sections as described previously [35,40]. Briefly, the eyes were enucleated, fixed, and embedded in paraffin. Serial sections (4 μm) of whole eyes were cut sagittally, through the cornea and parallel to the optic nerve and stained with hematoxylin and eosin. The preretinal neovascular cells nuclei were counted on eight discontinuous sections per eye and compared with the result of the 15-LOX-1 group, the OIR group, the GFP group, and the normal group.

### Real-time polymerase chain reaction

Real-time PCR was performed to detect the mRNA expression levels of 15-LOX-1 and VEGF-A. Retinas were separated from eyeballs (n=6) on an iced plate and immediately frozen in liquid nitrogen for further use. Total RNA was extracted from the frozen retinas tissue by using Trizol® Reagent (Invitrogen, Carlsbad, CA). cDNA synthesis was conducted according to the RNA PCR kit protocol (Takara, Dalian, China). β-actin was used as a normalizing control. The primer sequences were *15-LOX-1*: 5^’^-TTG GTT CTA CTG GGT TCC TAA TG-3^’^ (forward) and 5^’^-GGA GCC AAA CGA CAT TTA TCT G-3^’^ (reverse); *VEGF-A*: 5^’^-GCT ACT GCC GTC CGA TTG AG-3^’^ (forward) and 5^’^-GGC TTT GTT CTG TCT TTC TTT GG-3^’^ (reverse), *β-actin*: 5^’^-CTG AGA GGG AAA TCG TGC GT-3^’^ (forward) and 5^’^-CCA CAG GAT TCC ATA CCC AAG A-3′ (reverse). The PCR reaction was performed in a volume of 25 μl using SYBR Green Mix (Toyobo, Shanghai, China) on the Rotor-Gene 3000 Real-time PCR instrument (Corbett Research, Sydney, Australia). The thermal cycling program consisted of 1 min at 95 °C, 40 cycles of 15 s at 95 °C, 15 s at 58 °C, and 45 s at 72 °C. Relative quantiﬁcation of the gene expression was performed using the 2^(-ΔΔCT)^ method [44]. All experiments were performed in triplicate.

### Western blot analysis

Retinas were separated from eyeballs (n=8) in each group on an iced plate and immediately frozen in liquid nitrogen for further use. The retinas were homogenized in 200 μl of lysis buffer (20 mmol/l Tris, pH 7.4, 150 mmol/l NaCl, 1 mmol/l EDTA, 1 mmol/l orthovanadate, 1 mmol/l phenylmethylsulfonyl ﬂuoride, 1 μg/ml leupeptin, and 10 μg/ml aprotinin). After centrifugation at 12,000 × *g* for 5 min at 4 °C, the supernatant was collected. The protein concentration of the sample was determined using the Bradford assay. Equal amounts (40 μg) of proteins from each sample were loaded on sodium dodecyl sulfate PAGE (SDS–PAGE) and then transferred onto nitrocellulose membrane (Hybond-C, Amersham, Arlington Heights, IL) at 200 mA for 1 h. After blocking of nonspeciﬁc binding sites with 5% skim milk for 1 h, the membrane was incubated with the primary antibodies as the following dilutions: mouse monoclonal antibody to 15-LOX-1 at 1:500 (Abcam) and rabbit polyclonal antibody to VEGF-A at 1:300 (Bioss Biotechnology, Beijing, China) overnight at 4 °C. Antibody dilutions were made in a solution of 5% skim milk /0.1% Tris-buffered saline Tween-20. Then, membranes were washed with Tris buffered saline (TBS) and incubated with horseradish peroxidase–conjugated goat anti-mouse or rabbit immunoglobulin G secondary antibody (Boster Biotechnology, Wuhan, China) in blocking buffer for 1 h at room temperature. After three washes, the proteins were detected with the ECL kit (Amersham Pharmacia Biotech UK Ltd., Little Chalfont, UK). β-actin staining served as the internal standard for all membranes. Experiments were repeated three times.

### Statistical analysis

All values are given as means±standard deviation (SD). For comparisons between the normal group and the OIR group, we employed two-way ANOVA followed by the Bonferroni post-test. For comparisons among the normal group, the OIR group, the 15-LOX-1 group, and the GFP group, data were analyzed with one-way ANOVA followed by the Bonferroni post-test. P values less than 0.05 were considered statistically significant.

## Results

### Decreased 15-LOX-1 expression in the oxygen-induced ischemic retinopathy model

**Figure 1:**
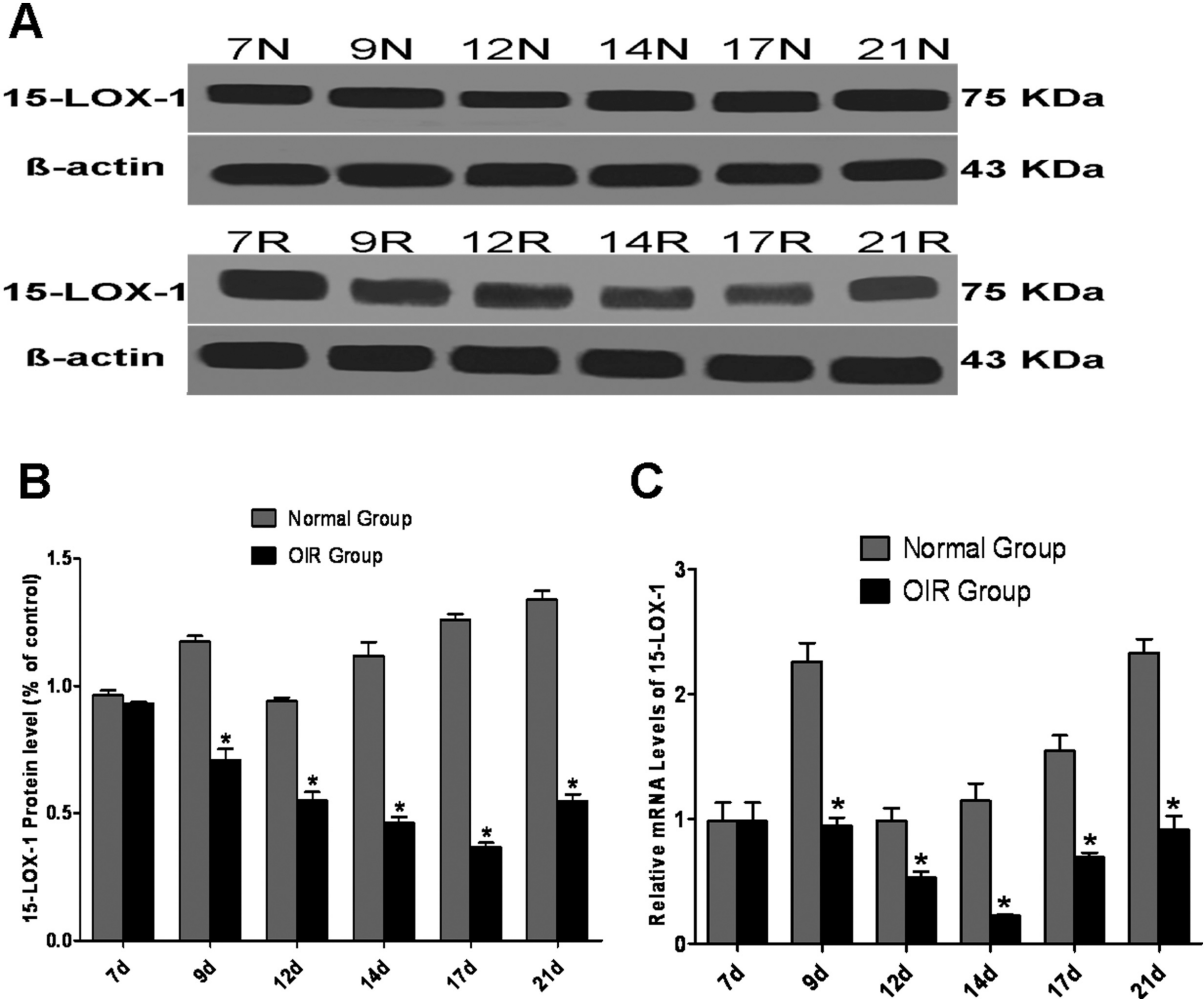
15-LOX-1 levels in the retina during the development of neovascularization. Western blot analysis of 15-LOX-1: retinas from four normal group and four oxygen-induced retinopathy (OIR) group mice were dissected at each time point as indicated. **A**: The same amount of retinal proteins from each mouse was blotted sequentially with the anti-15-LOX-1 and anti-β-actin antibodies. The blot shows results from normal group (N) and OIR group (R) mice. **B**: 15-LOX-1 protein relative amounts expression levels in normal group (N) and OIR group (R) mice. The bands were quantified with densitometry and normalized by β-actin. The levels of 15-LOX-1 protein in the OIR group were decreased compared with the normal group. Statistical significance was determined with two-way ANOVA (OIR group versus Normal group *p<0.05, n=3). **C**: 15-LOX-1 mRNA levels in the retina with neovascularization. The same amounts of total RNA from three normal group and three OIR group mice retinas at various ages were used for real-time PCR analysis. The levels were normalized with β-actin. The 15-LOX-1 mRNA levels in the OIR group were decreased compared with the normal group. Statistical significance was determined with two-way ANOVA (OIR group versus Normal group *p<0.05, n=3).

To determine whether 15-LOX-1 plays a role in RNV, we investigated the expression of 15-LOX-1 in the mouse model of OIR at different times, which was characterized by pathological RNV. 15-LOX-1 was measured with western blot analysis and normalized with β-actin. At P9 and P12, after exposure to 75% O_2_ for two and five days, the 15-LOX-1 protein levels in the OIR group were decreased by 40% and 42% more than those in the normal group. At P14 and P17 (two and five days after returning from hyperoxia), the protein levels of 15-LOX-1 in the OIR group decreased to 41% and 29% of the respective control levels. The lowest 15-LOX-1 level in the OIR group was observed at P17 when the most aggressive progression of RNV occurred. When RNV was essentially completed from P17 to P21, the 15-LOX-1 levels began to recover (Figure 1A, B). Real-time PCR analysis demonstrated that the changes in the expression of 15-LOX-1 occurred at the RNA levels in the OIR model. Changes in the 15-LOX-1 mRNA levels were detected before those of the protein levels. Decreased 15-LOX-1 mRNA was also detected as early as at P9, with the lowest 15-LOX-1 RNA level (approximately 20% of the control level) observed at P14, whereas the lowest 15-LOX-1 protein level was observed at P17. The 15-LOX-1 mRNA began to recover after P14 (Figure 1C). These results suggested that the decreased expression of 15-LOX-1 during RNV in OIR and the retinal 15-LOX-1 levels were negatively correlated with the progression of RNV.

### Overexpression of the 15-LOX-1 transgene in the mouse retina

**Figure 2:**
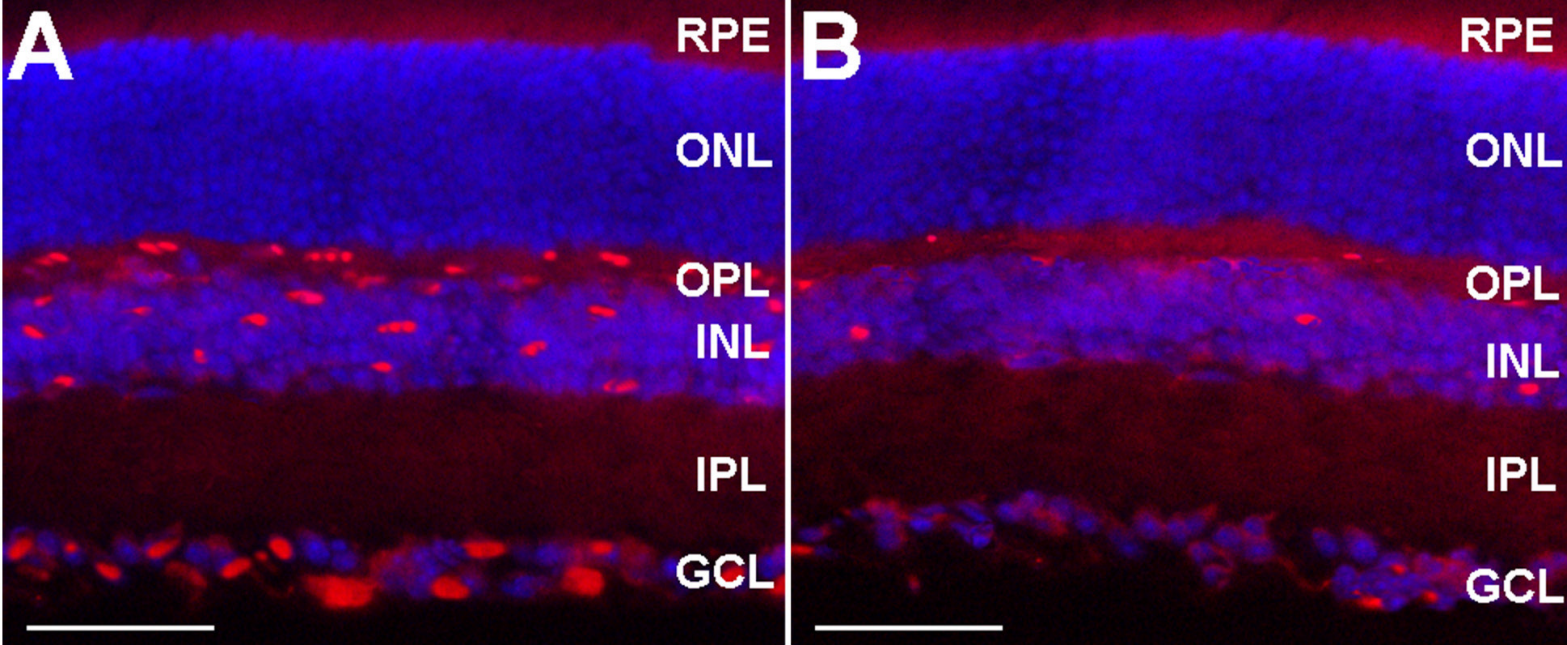
This figure shows the transgene expression of recombinant adenoviral vectors in the retinas of 12-day-old oxygen-induced retinopathy mice, five days after intravitreous injections of approximately 1.0×10^9^ plaque formation unit of *Ad-15-LOX-1* or *Ad-GFP* (four mice per experimental group). Immunofluorescence staining indicated that the 15-LOX-1 protein is more strongly expressed in retinas from the *Ad-15-LOX-1* group (**A**) mainly in the outer plexiform layer (OPL), inner nuclear layer (INL), and ganglion cell layer (GCL) than in retinas from the *Ad-GFP* group (**B**). Cell nuclei were stained with 4', 6-diamidino −2-phenylindole (DAPI). Images **A** and **B** were taken at 400× magnification for optimal comparison, scale bars=35 um.

To determine whether the 15-LOX-1 transgene is expressed in the mouse retina, 12-day-old OIR group mice received intravitreous injections of *Ad-15-LOX-1* or *Ad-GFP*. Mice were euthanized five days after intravitreous injections, and the retinas were dissected for further experiment. Immunofluorescence staining of retinal sections revealed 15-LOX-1 expression was clearly shown, primarily in the outer plexiform layer (OPL), inner nuclear layer (INL), and ganglion cell layer (GCL) retinas of the intravitreous injections of *Ad-15-LOX-1* mice (Figure 2A), whereas mice intravitreous injections of *Ad-GFP* showed a small amount of fluorescence in the OPL, INL, and GCL retinas (Figure 2B). In our control experiment omitting primary antibody (15-LOX-1) and secondary antibody, no positive staining was presented (data not shown).

**Figure 3:**
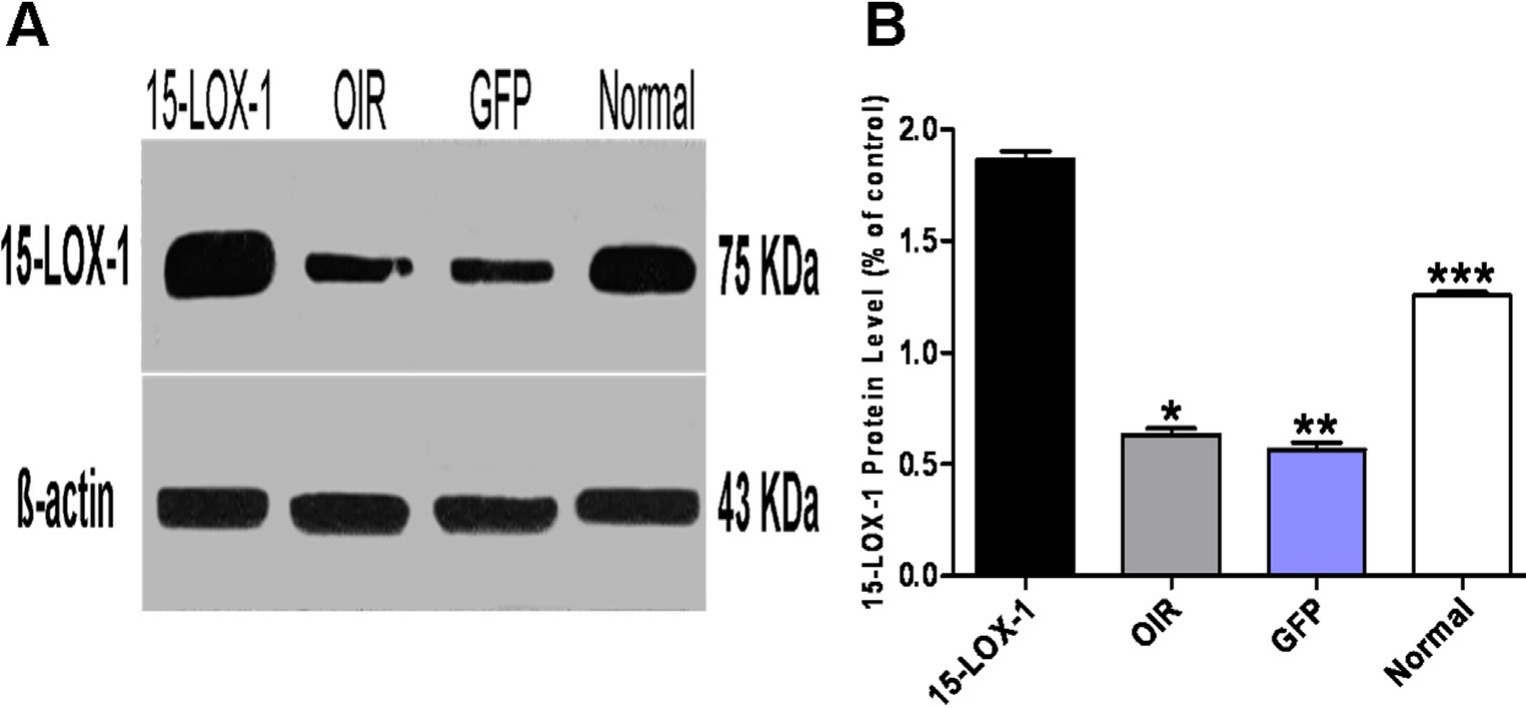
Western blot analysis confirmed overexpression of 15-LOX-1. The experiments were divided into the oxygen-induced retinopathy treated with adenoviral-15-lipoxygenase-1 group (the 15-LOX-1 group), the untreated oxygen-induced retinopathy group (the OIR group), the oxygen-induced retinopathy treated with adenoviral-green fluorescence protein group (the GFP group) and the normal control group (the normal group), with four mice per experimental group. Western blot analysis showed that the 15-LOX-1 protein levels were higher in the 15-LOX-1 group mice retinas than those in OIR group, GFP group, and normal group mice retinas. The bands were quantified with densitometry and normalized by β-actin; data were analyzed as means±SD (**A**, **B**). 15-LOX-1 group versus OIR group *p<0.05, n=3; 15-LOX-1 group versus GFP group **p<0.05, n=3; 15-LOX-1 group versus Normal group ***p<0.05, n=3.

**Figure 4:**
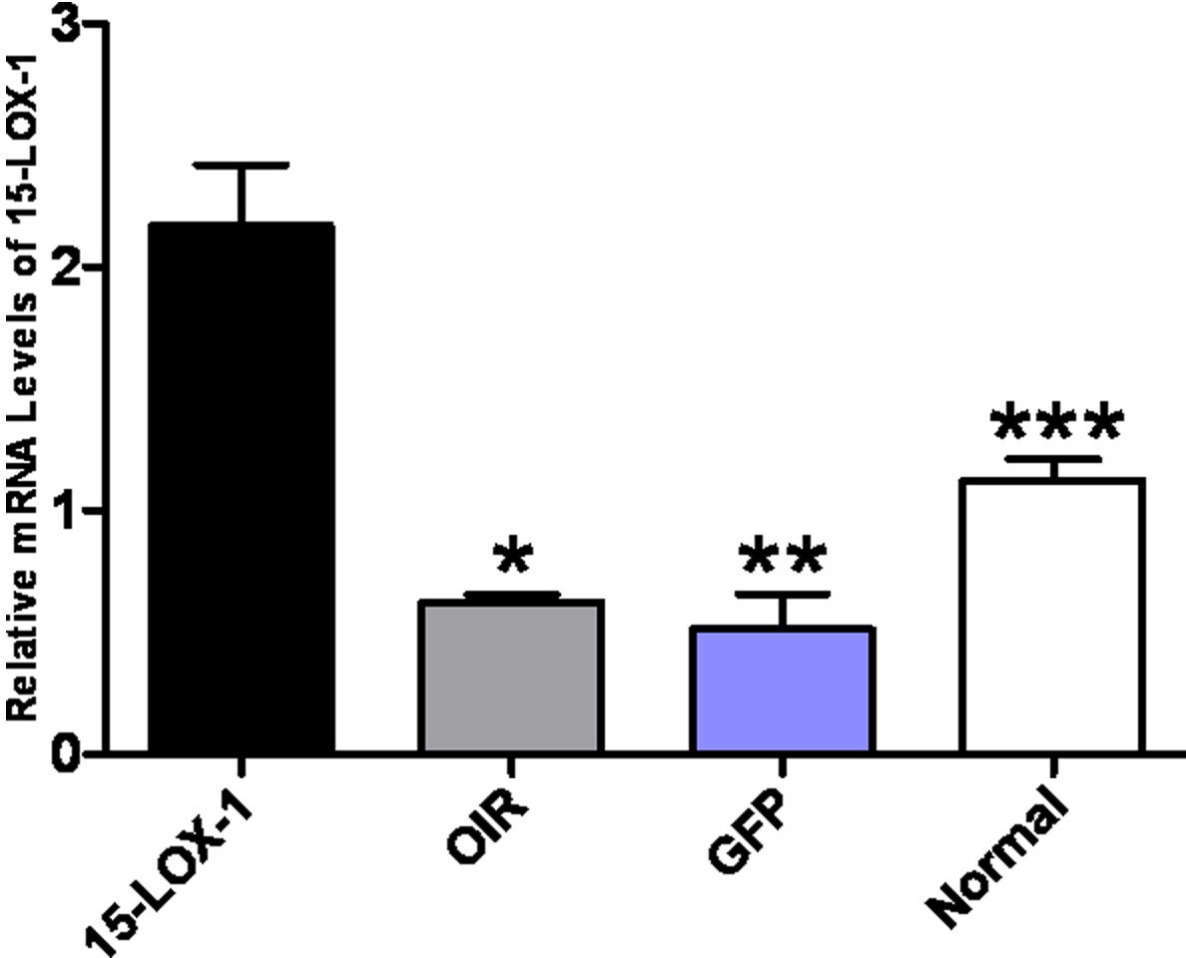
Real-time PCR analysis confirmed overexpression of 15-LOX-1. The experiments were divided into the oxygen-induced retinopathy treated with adenoviral-15-lipoxygenase-1 group (the 15-LOX-1 group), the untreated oxygen-induced retinopathy group (the OIR group), the oxygen-induced retinopathy treated with adenoviral-green fluorescence protein group (the GFP group) and the normal control group (the normal group), with three mice per experimental group. Real-time PCR analysis showed that the mRNA expression levels of 15-LOX-1 in the 15-LOX-1 group were higher than those in the OIR group, the GFP group, and the normal group. Data are shown as means±SD; the relative amount of mRNA was normalized to β-actin. 15-LOX-1 group versus OIR group *p<0.05, n=3; 15-LOX-1 group versus GFP group **p<0.05, n=3; 15-LOX-1 group versus Normal group ***p<0.05, n=3.

Western blot analysis showed that the 15-LOX-1 protein levels were substantially higher in the 15-LOX-1 group mouse retinas than those in the normal group (p<0.01, n=3), OIR group (p<0.01, n=3), and GFP group (p<0.01, n=3) mouse retinas (Figure 3A, B). Real-time PCR assays further confirmed that the mRNA expression levels of 15-LOX-1 in the 15-LOX-1 group were also higher than those in the normal group (1.94-fold, p<0.01, n=3), the OIR group (3.50-fold, p<0.01, n=3), and the GFP group (4.25-fold, p<0.01, n=3; Figure 4).

### Effects of 15-LOX-1 overexpression on oxygen-induced ischemic retinal neovascularization

**Figure 5:**
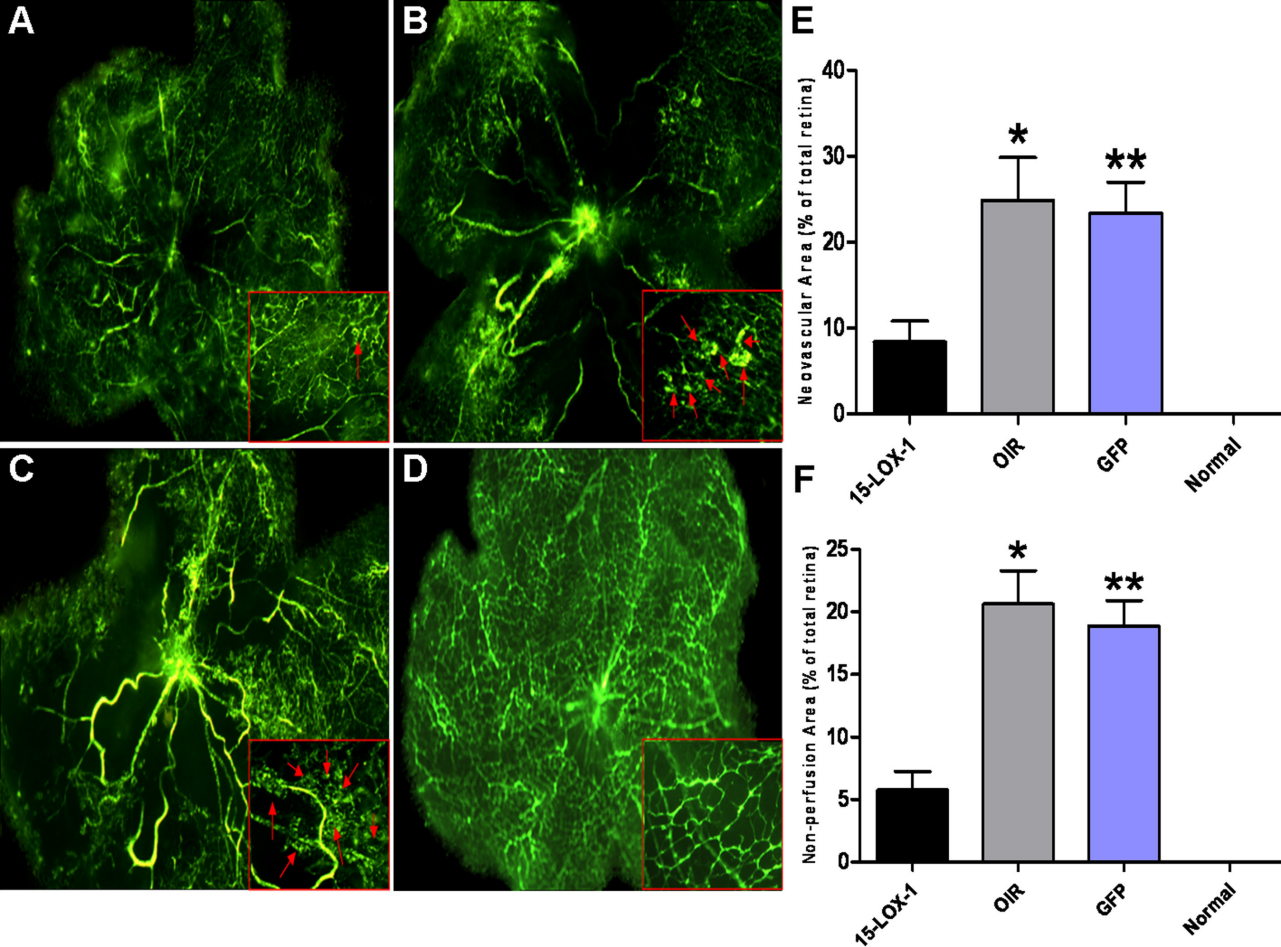
The inhibitory effect of 15-LOX-1 overexpression on retinal neovascularization in the oxygen-induced ischemic retinopathy model. The experiments were divided into the oxygen-induced retinopathy treated with adenoviral-15-lipoxygenase-1 group (the 15-LOX-1 group), the untreated oxygen-induced retinopathy group (the OIR group), the oxygen-induced retinopathy treated with adenoviral-green fluorescence protein group (the GFP group) and the normal control group (the normal group), with four mice per experimental group. All mice were perfused with fluorescein isothiocyanate-dextran at postnatal day 17 and the retina flatmounted. The images were representatives the retinal angiographs from the eyes of 15-LOX-1 group (**A**), OIR group (**B**), GFP group (**C**) and Normal group (**D**). The magnification of **A**-**D** is 40×. The red rectangles in each figure are the magnification of randomly selected foci of retina, and the red arrows indicate the neovascular tufts. **E**: Retinas neovascular areas were measured and compared with the 15-LOX-1 group, OIR group, GFP group and normal group mice. Retinal neovascularization was quantified by measuring the ratio of the neovascular tuft area to the total retinal area using Image-Pro Plus 6.0 software (means±SD, n=8), 15-LOX-1 group versus OIR group *p<0.05, n=8; 15-LOX-1 group versus GFP group **p<0.05, n=8. F: Retinas non-perfusion areas were measured and compared with 15-LOX-1 group, OIR group, GFP group and Normal group using Image-Pro Plus 6.0 software (means±SD, n=8), 15-LOX-1 group versus OIR group *p<0.05, n=8; 15-LOX-1 group versus GFP group **p<0.05, n=8.

The impact of 15-LOX-1 overexpression on oxygen-induced ischemic RNV was evaluated in the OIR model, which develops oxygen-induced ischemic RNV [35]. To determine whether overexpression of 15-LOX-1 suppresses oxygen-induced ischemic RNV, we examined the retinal vasculature of the 15-LOX-1 group, the OIR group, the GFP group, and the normal group using fluorescein angiography in retinal flatmounts at P17. The results revealed significant neovascularization in the flatmounted retinas from the OIR group and GFP group mice (Figure 5B,C). In contrast, the retinas from the 15-LOX-1 group developed less severe neovascular tufts (Figure 5A). Normal group mice maintained in room air showed no neovascularization in the retinal vasculature (Figure 5D). RNV was quantified by measuring areas of neovascular tufts in the retinal whole mounts, which showed that the retinas from the 15-LOX-1 group mice had significantly smaller retinal neovascular tuft areas, approximately 34% of those in the OIR group and 36% in the GFP group (Figure 5E). To further determine whether overexpression of 15-LOX-1 ameliorates the extent of hyperoxia-mediated vasoobliteration in OIR, we measured the non-perfusion areas in the central retina at P17. In the 15-LOX-1 group, the non-perfusion areas were significantly smaller than those in the OIR group (p<0.05, n=8) and the GFP group (p<0.05, n=8; Figure 5F). In addition, we found no retinal toxicity and no retinal detachment or other damage related to the needle puncture. Taken together, our results suggest that 15-LOX-1 overexpression ameliorated hyperoxia-mediated vasoobliteration and inhibited hypoxia-induced RNV.

### Reduced preretinal neovascularization in oxygen-induced ischemic retinopathy

**Figure 6:**
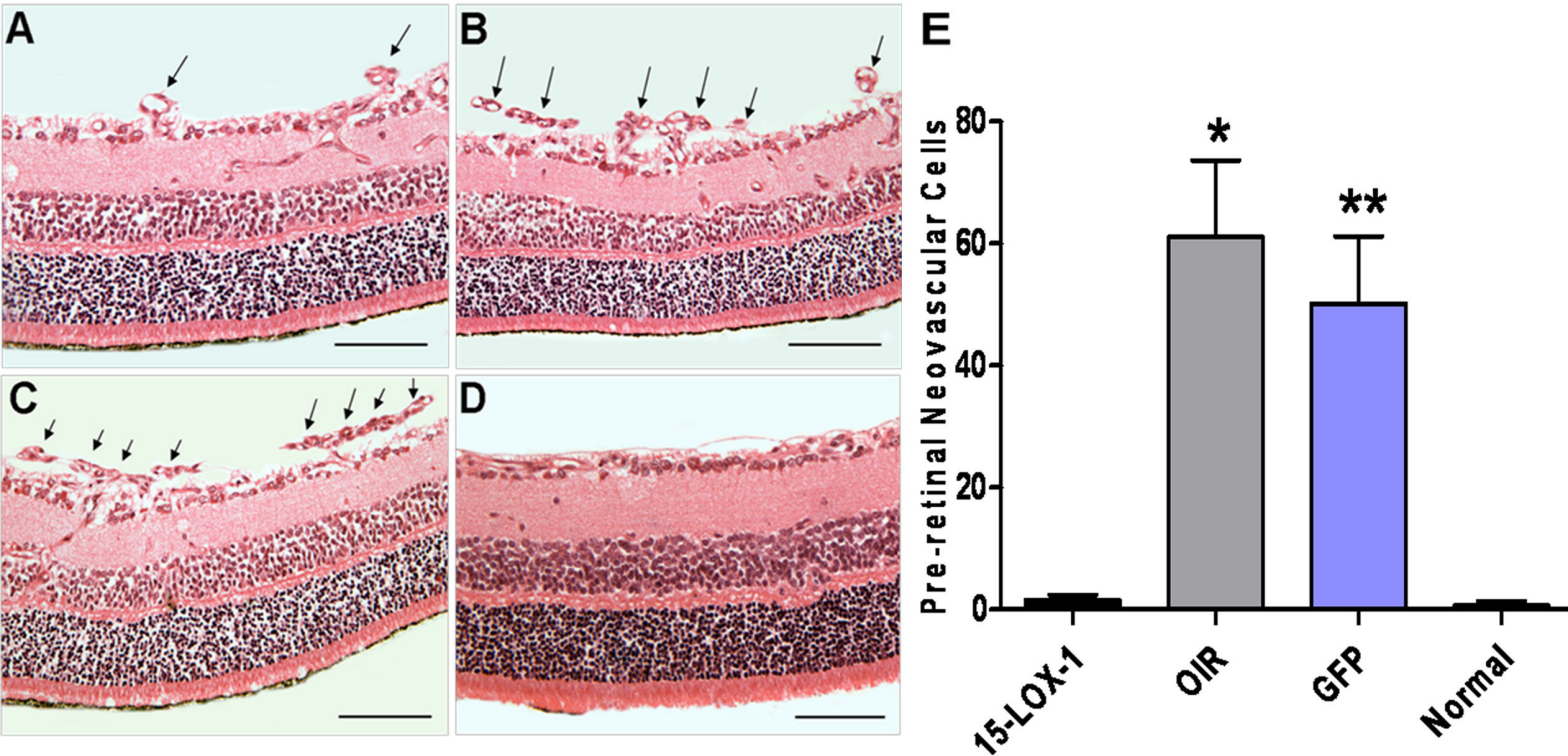
The effect of 15-LOX-1 overexpression on preretinal neovascularization in mice with oxygen-induced ischemic retinopathy. The experiments were divided into the oxygen-induced retinopathy treated with adenoviral-15-lipoxygenase-1 group (the 15-LOX-1 group), the untreated oxygen-induced retinopathy group (the OIR group), the oxygen-induced retinopathy treated with adenoviral-green fluorescence protein group (the GFP group) and the normal control group (the normal group), with eight mice per experimental group. At postnatal day 17, all mice were fixed, sectioned, and stained with hematoxylin and eosin. Preretinal neovascular cells were counted on eight non-continuous sections per eye and averaged. The images were representatives the retinal sections from the eyes of 15-LOX-1 group (**A**), OIR group (**B**), GFP group (**C**) and Normal group (**D**). Arrows indicate preretinal neovascular cells. The magnification of **A**-**D** is 200×. Scale bars=50 um. **E**: The average numbers of preretinal neovascular cells (means±SD, n=8) of the 15-LOX-1 group mice were compared with the OIR group, GFP group, and normal group mice using one-way ANOVA. 15-LOX-1 group versus OIR group *p<0.05, n=8; 15-LOX-1 group versus GFP group **p<0.05, n=8.

To further confirm the effect of 15-LOX-1 overexpression on RNV, we quantified preretinal neovascular cells, a characteristic feature of the OIR model [35,45]. The preretinal neovascular cells growing into the vitreous humor were counted on eight non-continuous cross-sections from each eye following an established method [35,40]. The preretinal neovascular cells nuclei were counted if they were found on the vitreous cavity side of the internal limiting membrane on eight discontinuous sections per eye, and cross-sections that included the optic nerve were excluded. As shown in Figure 6A-D, the number of preretinal neovascular cells in the retinas from the 15-LOX-1 group (1.50±0.93) was obviously lower than those in the retinas from the OIR group (61.13±12.53, p<0.05, n=8) and the GFP group (50.13±11.03 p<0.05, n=8), confirming the antineovascularization effect of 15-LOX-1 overexpression in the retina.

### Overexpression of 15-LOX-1 suppresses overproduction of vascular endothelial growth factor-A in the oxygen-induced ischemic retinopathy model

**Figure 7:**
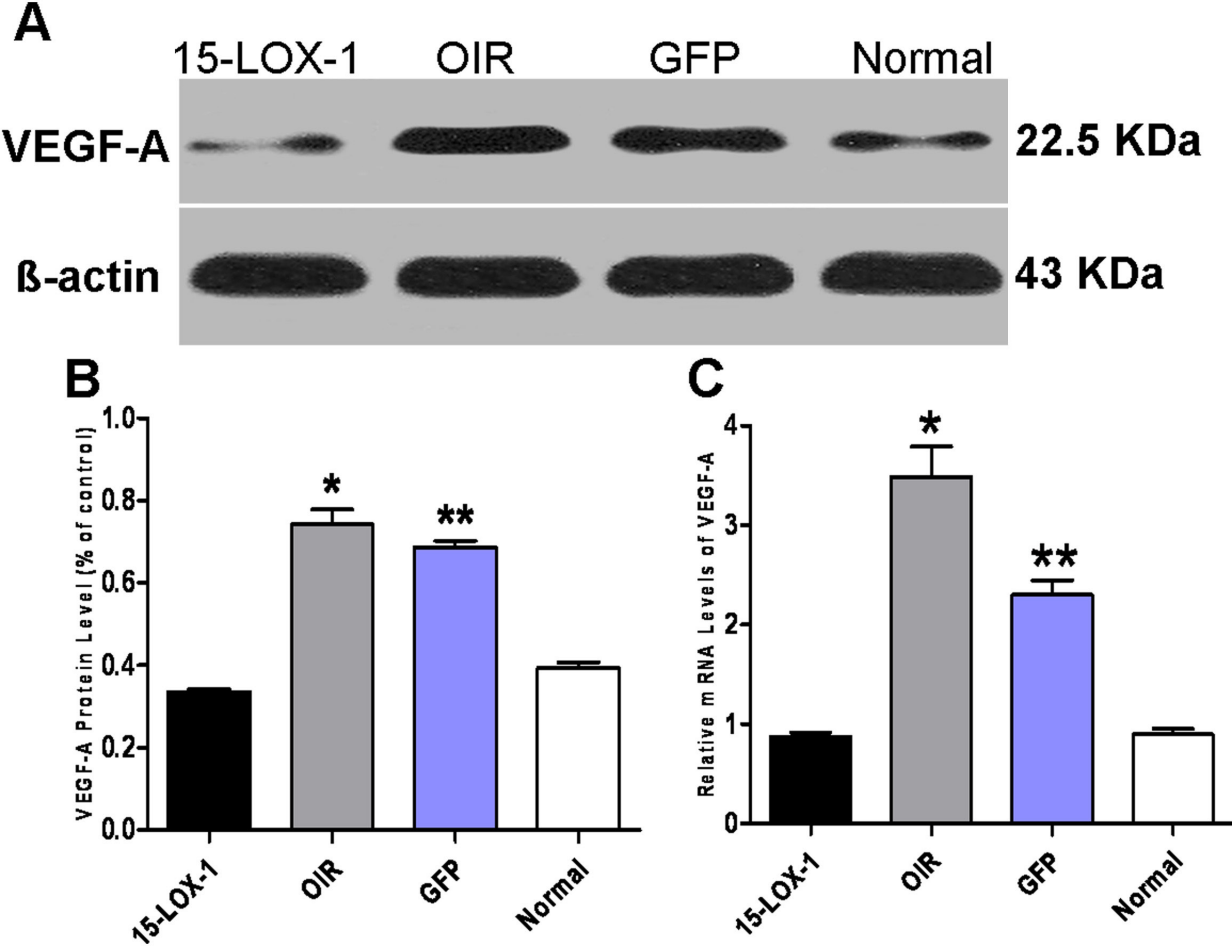
The effects of 15-LOX-1 overexpression on the retinal levels of VEGF-A in oxygen-induced ischemic retinopathy mice. The experiments were divided into the oxygen-induced retinopathy treated with adenoviral-15-lipoxygenase-1 group (the 15-LOX-1 group), the untreated oxygen-induced retinopathy group (the OIR group), the oxygen-induced retinopathy treated with adenoviral-green fluorescence protein group (the GFP group) and the normal control group (the normal group). All mice were euthanized, and the eyes were enucleated at postnatal day 17. **A**: Retinal levels of VEGF-A and β-actin were measured with western blot analysis (four mice per experimental group). The expression levels of VEGF-A protein were quantified with densitometry and normalized to β-actin. **B**: The expression of VEGF-A (means±SD, n=3) was compared for the 15-LOX-1 group, the OIR group, the GFP group, and the normal group using one-way ANOVA (15-LOX-1 group versus OIR group *p<0.05, n=3; 15-LOX-1 group versus GFP group **p<0.05, n=3). **C**: Real-time PCR analysis of VEGF-A expression showed that the mRNA expression levels of VEGF-A in 15-LOX-1 group were lower than those in the OIR group and the GFP group (three mice per experimental group, one-way ANOVA). The relative amount of mRNA was normalized to β-actin (means±SD, n=3). 15-LOX-1 group versus OIR group *p<0.05, n=3; 15-LOX-1 group versus GFP group **p<0.05, n=3.

To investigate the inhibitory mechanism of 15-LOX-1 overexpression during RNV in OIR, we compared the expression levels of VEGF-A in all groups. As shown with western blot analysis, the protein levels of VEGF-A were decreased in the 15-LOX-1 group compared with the OIR group (p<0.05, n=3) and the GFP group (p<0.05, n=3; Figure 7A, B). Moreover, real-time PCR showed that the mRNA levels of VEGF-A were significantly lower in the 15-LOX-1 group compared with the OIR group (p<0.05, n=3) and the GFP group (p<0.05, n=3; Figure 7C). Taken together, these results suggested that 15-LOX-1 overexpression has an inhibitory effect on RNV in the OIR model via downregulation of VEGF-A expression.

## Discussion

RNV occurs in several ischemic retinopathies, including age-related macular degeneration, proliferative diabetic retinopathy, and retinopathy of prematurity [46], which could lead to blindness if left untreated. For many years, treatment for RNV in ocular diseases was limited to laser and cryosurgery of the avascular zone, which, although effective for reducing severe vision loss, could lead to serious side effects. It is important to develop new therapies for pathological angiogenesis. In recent years, progress has been made in the pharmacological inhibition of proangiogenic factors, VEGF. In this regard, it is important that anti-VEGF strategies involving intravitreal injections have recently emerged as valuable new therapies for ocular neovascularization diseases [47-50].

15-LOX-1 has been implicated in angiogenesis, but previous studies were apparently contradictory on the angiogenic properties of 15-LOX-1, which varied from promoting an angiogenic effect [51] to suppressing angiogenesis [52-54]. The levels of 15-LOX-1 in normal and pathological conditions have not been revealed previously, and the role of 15-LOX-1 in oxygen-induced ischemic RNV needs further investigation. To the best of our knowledge, this is the first study to describe changes in the expression of 15-LOX-1 during RNV in OIR. The major findings of our study include the following: 1) decreased expression of 15-LOX-1 during RNV in OIR, 2) retinal 15-LOX-1 levels were negatively correlated with the progression of RNV in OIR, and 3) inhibition of VEGF-A expression and RNV in OIR by overexpression of 15-LOX-1.

The mouse model of OIR is a widely used animal model of oxygen-induced ischemic RNV [35,45]. In this model, pathological NV presents in two phases: the hyperoxia-mediated vasoobliteration phase and the hypoxia-induced NV phase [43]. In the first phase, hyperoxia causes vessel regression in the central retina and the cessation of normal radial vessel growth, which inhibits retinal vessel growth and causes significant vessel loss. Regarding the mechanisms involved in this phase, it is well established that hyperoxia suppresses VEGF [55,56], which leads to endothelial cell apoptosis [43,57]. In the present study, we demonstrated that decreased mRNA and protein expression of 15-LOX-1 occurred during the hyperoxia-mediated vasoobliteration phase. We speculate that decreased 15-LOX-1 expression via downregulated VEGF expression induced capillary endothelial cell apoptosis under hyperoxia conditions. In the second phase, NV developed between P12 and P17. Our studies demonstrated decreased 15-LOX-1 expression in this model. However, the regulatory mechanism of 15-LOX-1 expression is presently unclear. Our real-time PCR and western blot analysis detected decreased 15-LOX-1 mRNA levels and protein levels, respectively, in the retina with hypoxia-induced NV. Moreover, the decreased 15-LOX-1 mRNA levels were approximately to the same extent as but preceded the protein level changes. These results seem to support regulation at the RNA level. Furthermore, the time course of the 15-LOX-1 change was negatively correlated with the development and progression of RNV. These findings suggest that 15-LOX-1 may act as a negative regulator of angiogenesis in the eye, and decrease 15-LOX-1 levels leading to RNV.

To further study the role of 15-LOX-1 in vivo under different conditions, we investigated 15-LOX-1 expression under normoxic conditions; high levels of 15-LOX-1 in the retina do not affect normal vascular development. Under pathological conditions, the retina has decreased expression of 15-LOX-1, but interestingly, overexpression of 15-LOX-1 inhibited pathological NV. The mechanism(s) for these differential effects of 15-LOX-1 on normal vascular formation and pathological NV remain(s) to be further investigated.

To evaluate the effect of 15-LOX-1 overexpression on RNV in the OIR mouse model, we used fluorescein angiography on retinal whole mounts. The results showed that oxygen-induced ischemic RNV, as quantified with neovascular tufts and preretinal neovascular cells, was suppressed in the 15-LOX-1 group compared with those in the OIR group and the GFP group. Our study presents the first evidence suggesting that overexpression of 15-LOX-1 in the retina inhibits oxygen-induced ischemic RNV. These observations further indicate that 15-LOX-1 functions as an antiangiogenic factor in the retina.

VEGF-A is an important growth factor in the pathogenesis of angioproliferative changes in the eye [58]. Several pieces of evidence indicated that VEGF-A stimulated pathological angiogenesis by signaling through VEGF receptors in a dose-dependent manner and was upregulated by ischemia [23,59]. However, few studies have elucidated the relationship between VEGF-A and 15-LOX-1 on RNV in the OIR model. In the present study, intravitreal 15-LOX-1 overexpression efﬁciently inhibited RNV by reducing VEGF-A mRNA and protein expression. These results conﬁrm that overexpression of 15-LOX-1 can exert an antiangiogenic effect by decreasing VEGF-A production. The latest research showed 15-LOX-1 metabolites: 15-(S)-HETE promoted angiogenesis via upregulation of VEGF expression while 15-(S)-HPETE inhibited angiogenesis via downregulation of VEGF expression [20]. We speculate that the expression levels of 15-LOX-1 can regulate the rate of conversion of 15-(S)-HPETE to 15-(S)-HETE in a manner dependent on the cellular level of peroxidases that catalyze the reduction of 15-(S)-HPETE to 15-(S)-HETE, and overexpression of 15-LOX-1 inhibits 15-(S)-HPETE converting to 15-(S)-HETE in the mouse model of OIR. Further experiments must be conducted to research the levels of expression of 15-(S)-HPETE and 15-(S)-HETE during RNV in the mouse model of OIR, and explore the underlying regulating mechanisms involving 15-LOX-1 and its metabolites, 15-(S)-HPETE and 15-(S)-HETE in OIR.

In summary, we have shown that decreased expression of 15-LOX-1 in OIR is negatively correlated with the progression of RNV, and overexpression of 15-LOX-1 prevents RNV. The mechanism for the inhibition involves the 15-LOX-1/VEGF-A balance disruption. Further investigation is required to identify the mechanism(s) of how 15-LOX-1 modulates VEGF-A expression at different pathological stages of RNV.
